# Vaginal Endosalpingiosis: A Case Report and Literature Review

**DOI:** 10.7759/cureus.22949

**Published:** 2022-03-08

**Authors:** Ahmed Sabri, Margarita Loxas, Linnea Banker, Kevin Zhang, Wayne Penka

**Affiliations:** 1 Pathology and Laboratory Medicine, Creighton University School of Medicine, Omaha, USA

**Keywords:** gynecology, vagina, gynecologic pathology, benign, ectopic epithelium, vaginal endosalpingiosis, endosalpingiosis

## Abstract

Endosalpingiosis is a benign condition with unclear pathogenesis and clinical significance and is defined as the presence of ectopic fallopian tube-like epithelium. It can be found in multiple locations, most commonly in the pelvic peritoneum covering the ovaries, uterus, and fallopian tubes, and less commonly found in the lymph nodes, omentum, appendix, cervix, vulva, or vagina.

It is difficult to distinguish from endometriosis by gross appearance or localization, and theories propose that tissues of the secondary Mullerian system may undergo a metaplastic transformation, for example, from endosalpingiosis to endometriosis, which contributes to the debated association of endosalpingiosis with chronic pelvic pain. Additionally, there is evidence demonstrating a close association with reproductive tract neoplasms.

We report the clinical course, diagnosis including pathology, follow-up, and the treatment plan of vaginal endosalpingiosis in a 34-year-old woman presenting with a chronic painful right-sided vaginal mucosal ulceration, dyspareunia, and foul-smelling vaginal discharge. To our knowledge, this is the second reported case of vaginal endosalpingiosis and the first case with this presentation.

## Introduction

Endosalpingiosis (ES) is, by definition, the presence of ectopic fallopian tube-like epithelium [1]. Distinct from endometriosis, ES is characterized by ciliated glandular epithelium, absent endometrial stroma, and usually no inflammatory component [2]. It is most commonly encountered in the ovary and is rarely seen in areas such as the myometrium or the pelvic peritoneum. The pathogenesis and clinical significance of ES are ambiguous, as ES has been widely assumed to be an incidentally discovered and benign condition since it was first described in 1930. However, in recent years, there is growing evidence of the potential of increased risk for malignancy in patients with ES [3]. As a result, it is imperative to grow familiar with the features of this diagnosis. Here, we present a case of a woman with a five-year history of vaginal ulceration and foul-smelling discharge. The tissue displayed ciliated tubal epithelium on biopsy and the patient was given the diagnosis of vaginal ES.

## Case presentation

A 34-year-old woman presented with a right-sided vaginal mucosal ulceration as well as a foul-smelling vaginal discharge for the last five years. The ulceration was painful to palpation and resulted in a constant general pelvic discomfort. She also reported pain with sexual intercourse, describing the pain as burning and rubbing sensation. She denied any bleeding between menstrual cycles, post-coital bleeding, or bowel or bladder symptoms.

The patient had been previously diagnosed with recurrent bacterial vaginosis with no resolution of symptoms with treatment. She had a history of three vaginal deliveries, one of which, in 2008, required forceps assistance and resulted in a fourth-degree vaginal laceration. The injury was repaired with a suture. The patient subsequently underwent MRI to assess for rectovaginal fistula, which did not show evidence of anatomic abnormality or further injury. She did not have a history of sexually transmitted infections (STIs). Upon physical exam, a 3 cm vaginal ulceration of the right vaginal sidewall was noted. It was described to be located about 3 cm from the vaginal introitus. The ulcer was described as having a puckered and thickened border, which was exquisitely tender to palpation and bled when touched. There was a small area of beefy-red granulation tissue present near the ulcer. The lesion did not have the appearance of necrosis or acute infection. A large ectropion and several nabothian cysts of the cervix were also visualized.

**Figure 1:**
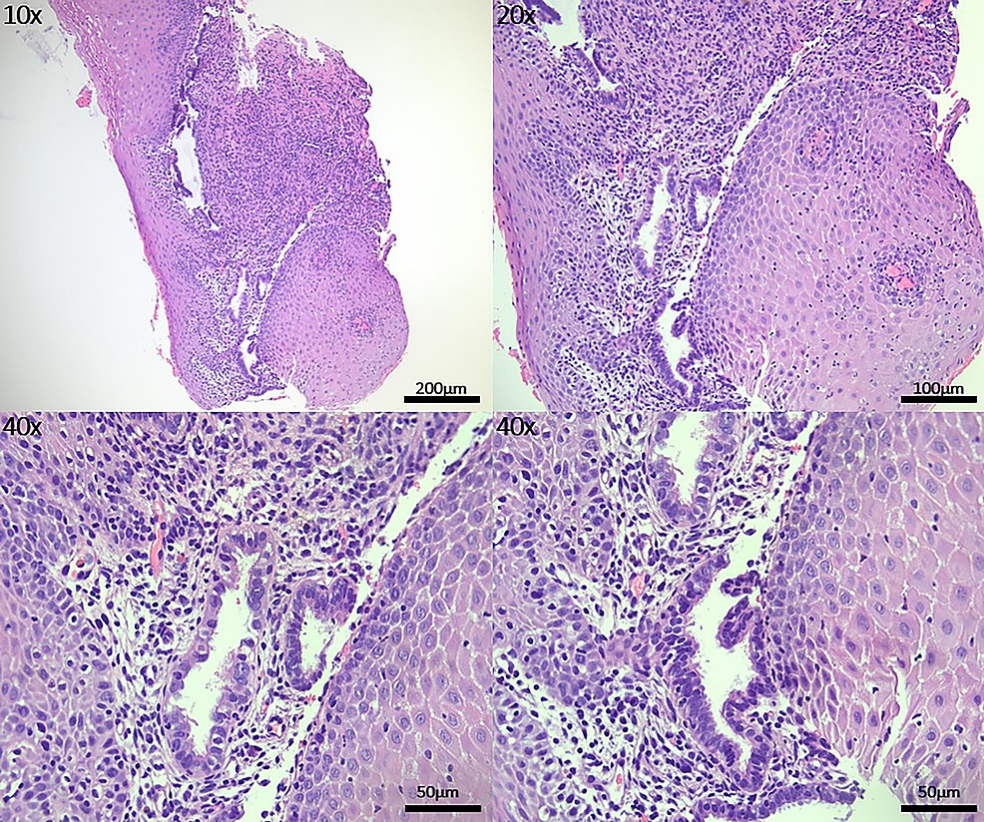
Right vaginal lesion biopsy on the hematoxylin and eosin (H&E) stains. One area pictured from lower magnification (10x) to higher magnification (40x) showed ectopic glands lined by fallopian tube ciliated epithelium. At the higher magnification (40x), three types of cells can be appreciated: ciliated columnar, non-ciliated columnar, and intercalary cells.

**Figure 2:**
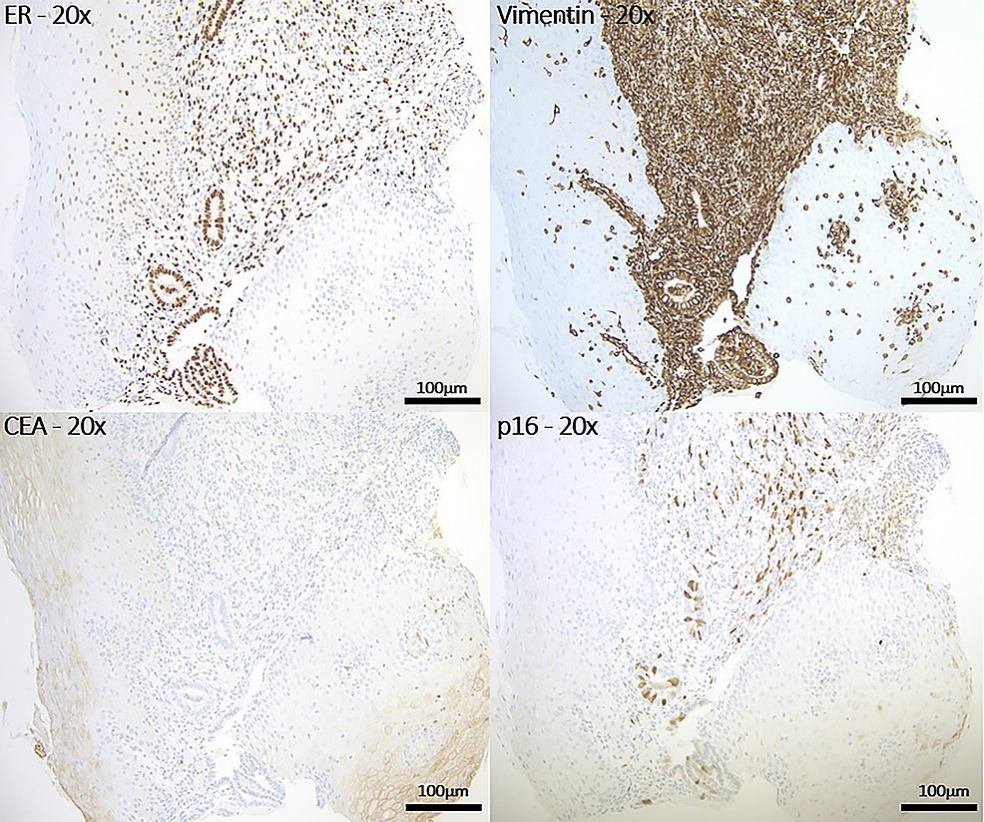
Right vaginal lesion biopsy on the immunohistochemical stains. Glands are strongly positive for estrogen receptor (ER) and vimentin stains. Carcinoembryonic antigen (CEA) stain is negative. Glands are showing patchy p16 staining. The findings are consistent with the normal staining pattern of benign tubal epithelium.

Anaerobic and aerobic cultures were obtained. The area was then anesthetized. When analgesia was achieved, a Tischler forceps was used to obtain the biopsy. It is also important to mention that the exam and biopsy were somewhat limited due to the anatomical location and tenderness of the lesion. For the pathology (Figures 1, 2), the sections from the vaginal lesion biopsy showed ectopic glands lined by fallopian tube ciliated epithelium with three types of cells: ciliated columnar, non-ciliated columnar, and intercalary cells. Those glands were strongly positive for estrogen receptor (ER) and vimentin stains, with patchy p16 staining consistent with the normal staining pattern of benign tubal epithelium. Carcinoembryonic antigen (CEA) stain was negative.

The patient was referred to the gynecologic oncology service for further workup, which was negative for STI, cervical dysplasia, and rectovaginal fistula. The patient will undergo wide local excision of the lesion with the hope of symptomatic improvement.

## Discussion

ES was first described in 1930 as “misplaced Mullerian mucosa…from the stump of the truncated fallopian tube” based on the local proliferative and invasive nature of tubal mucosa following salpingectomy [4]. True to the initial description, ES is histologically defined as the presence of ectopic tubal epithelium containing three cell types: ciliated glandular epithelium, non-ciliated mucous secreting cells, and intercalary or peg cells [5,6]. The histological findings are differentiated from those of endometriosis by the presence of ciliated glandular epithelium as well as a lack of endometrial stroma and inflammatory components [7]. Macroscopic features include simple cysts or complex papillary structures containing psammoma bodies [8]. ES is generally not hemorrhagic, unlike hormone-responsive endometriosis [6].

ES can be found in multiple locations, most commonly in the pelvic peritoneum covering the ovaries, uterus, and fallopian tubes [3]. It is less commonly found in the lymph nodes, omentum, appendix, cervix, vulva, or vagina. It is unclear why certain sites are more susceptible to the ectopic tubal epithelium, but proximity to the fallopian tube may be a factor.

Two main mechanisms giving rise to ES have been theorized: multifocal metaplastic process arising from peritoneal cells [9] or peritoneal implantation of sloughed tubal epithelial hyperplasia [10]. Regardless of the mechanism, ES is highly associated with chronic pelvic inflammatory insult or prior intrapelvic surgery [10]. Surgical intervention on the fallopian tubes carries a theoretical risk of ectopically seeding tubal epithelial cells. One study found nearly 80% of patients with ES had a prior history of tubal ligation or intrapelvic surgery [3].

The mean age at diagnosis is 43 years [5]. A recent study found 40% of cases occur in postmenopausal women, which was previously unrecognized [11]. ES is found in 1.4% to 12.5% of women undergoing laparoscopy for a variety of gynecological symptoms [3,12,13]. The high variability in incidence may be related to patient selection and tissue sampling in each study. Intraoperative presentation ranges from peritoneal nodular changes to omental or pelvic masses [14]. The heterogeneous appearance may mimic peritoneal tuberculosis or metastases of ovarian cancer [15] and is also difficult to distinguish from endometriosis by gross appearance or localization [5]. Diagnosis is rarely made preoperatively, but disseminated pelvic calcifications can sometimes be found by radiologic imaging [16,17].

Some studies have reported symptomatic pain in up to one-third of patients [18,19], while others have concluded that ES presents asymptomatically [5,20]. The debated association with chronic pelvic pain is complicated by the high concordance of ES with endometriosis, which ranges from 4.4% to 67% [3,12]. Interestingly, one theory proposes that tissues of the secondary Mullerian system may undergo a metaplastic transformation, for example, from ES to endometriosis [21]. Thus, further examination of this relationship may demonstrate whether ES is an independent risk for pain or infertility. While there is an established clinical correlation between endometriosis and infertility, existing literature reports either low or no association between ES and infertility [3,11].

While ES has been hypothesized to be an incidental intraoperative finding, there is evidence demonstrating a close association with reproductive tract neoplasms. Of particular importance is the increased association between premenopausal women with ES and gynecologic malignancy [11,22]. Gynecologic malignancy is found in nearly half of patients with ES [11], most commonly ovarian serous neoplasm (21%) and cervical cancer (18%) [3]. A large retrospective study found a significant increase of both uterine and ovarian cancer, but not cervical cancer, in patients with ES [3]. This study confirmed prior reported association with ovarian cancers [23-26]. Of interest is the newly found association with uterine cancer, which may further strengthen the hypothesis that tissues sharing a Mullerian origin have the capacity to undergo metaplastic conversion to other types of glandular epithelium that predispose to neoplastic transformation.

In terms of the literature, another case of vaginal ES is previously reported by Câmara et al. [27]. The patient presented with intermenstrual bleeding for several months without any suggestive history [27]. The physical examination revealed a soft, polypoid, and hemorrhagic neoplasm in the posterior vaginal cuff [27]. The patient underwent polypectomy with electrocoagulation, the tissue was sent to pathology and revealed vaginal ES, and the subsequent follow-ups did not show any new symptoms [27]. In our case, the patient had a different clinical presentation for the same underlying etiology. She presented with a painful vaginal ulcer with dyspareunia and foul-smelling vaginal discharge, and the pathology showed similar findings to the previously reported case of ES. Our patient will undergo wide local excision of the lesion with the hope of symptomatic improvement.

## Conclusions

To the authors’ knowledge, this is a rare case of vaginal ES with unique clinical features and gross findings. This is still a poorly understood entity and provides a challenging diagnosis to pathologists. Our aim is to report this rare entity in the literature in hopes of establishing a better understanding of the pathophysiology, clinical course, and burden on patients.
